# Performances of two different panfungal PCRs to detect mould DNA in formalin-fixed paraffin-embedded tissue: what are the limiting factors?

**DOI:** 10.1186/s12879-014-0692-z

**Published:** 2014-12-18

**Authors:** B Babouee Flury, M Weisser, S Savič Prince, L Bubendorf, M Battegay, R Frei, D Goldenberger

**Affiliations:** Division of Infectious Diseases and Hospital Epidemiology, University Hospital Basel, Petersgraben 4, Basel, 4031 Switzerland; Division of Pathology, University Hospital Basel, Schönbeinstrasse 40, Basel, 4031 Switzerland; Division of Clinical Microbiology, University Hospital Basel, Petersgraben 4, Basel, 4031 Switzerland

**Keywords:** Panfungal PCR, Internal transcribed spacer (ITS), Invasive fungal disease, Formalin-fixed paraffin-embedded tissue, Analytical sensitivity, Beta-globin gene

## Abstract

**Background:**

Detection of fungal DNA from formalin-fixed, paraffin-embedded (FFPE) tissue is challenging due to degradation of DNA and presence of PCR inhibitors in these samples. We analyzed FFPE samples of 26 patients by panfungal PCR and compared the results to the composite diagnosis according to the European Organization for Research and Treatment of Cancer (EORTC) criteria. Additionally we analyzed the quality of human and fungal DNA and their level of age-dependent degradation, as well as the existence of PCR inhibition in these tissue samples.

**Methods:**

We evaluated two 45-cycle panfungal PCR tests that target the internal transcribed spacer 2 (ITS2) as well as the ITS1-5.8S-ITS2 (ITS1-2) region. The PCRs were applied to 27 FFPE specimens from 26 patients with proven invasive fungal disease (IFD), and one patient with culture and histologically negative but PCR-positive fungal infection collected at our institution from 2003 to 2010. Quality of DNA in FFPE tissue samples was evaluated using fragments of the beta-globin gene for multiplex PCR, inhibition of PCR amplification was evaluated by spiking of *C. krusei* DNA to each PCR premix.

**Results:**

In 27 FFPE samples the ITS2 PCR targeting the shorter fragment showed a higher detection rate with a sensitivity of 53.8% compared to the ITS1-2 fragment (sensitivity 38%). Significant time-dependent degradation of human DNA in FFPE sample extracts was detected based on partial beta-globin gene amplification which was not in correlation to successful panfungal PCR identification of fungal organisms. The analytical sensitivity of both assays compared with culture was 60 CFU/ml of a *Candida krusei* reference strain. The performance of the two tests in an *Aspergillus* proficiency panel of an international external quality assessment programme showed considerable sensitivity.

**Conclusion:**

Panfungal diagnostic PCR assays applied on FFPE specimens provide accurate identification of molds in highly degraded tissue samples and correct identification in samples stored up to 7 years despite sensitivity limitations, mainly caused by partial PCR inhibition and DNA degradation by formalin.

## Background

Invasive fungal disease (IFD), especially in case of delayed initiation of appropriate antifungal treatment, is associated with severe morbidity and high mortality in immunocompromised, critically-ill patients [1]. Early and precise identification of fungal pathogens is crucial for appropriate management of patients with IFD as typical clinical manifestations often lack [2]. Despite detection of fungal elements in histological samples, fungal cultures from tissue biopsies often remain negative [2],[3]. An appealing alternative for culture-independent identification of the fungal agent might be panfungal PCR performed retrospectively from FFPE tissue. However, isolating high-quality DNA from FFPE biopsies is difficult because of the DNA degrading activity of the routinely used fixative formalin. Efficiency of DNA amplification from FFPE material also depends on the time period for fixation [4],[5].

The two fungal internal transcribed spacer regions 1 (ITS1) and 2 (ITS2) are highly variable and can be amplified by PCR using broad-range panfungal primers detecting potentially all fungal organisms [6]. The sequence analysis followed by comparison with reference sequences in databases enables the identification of the etiologic organism in many cases to the species level. The detection rates of different panfungal PCR approaches in FFPE tissue specimens compared with conventional techniques have been reported in previous studies to be 51% to 62.7% [7]-[12]. In the study of Paterson et al. [10] the molecular detection of the fungal pathogens could be raised to 93% when combining PCR with hybridization. However storage time to of the FFPE specimens was not specified in many of the studies mentioned [8],[9],[11],[12].

The aim of the present study was the evaluation of two diagnostic panfungal PCR detection systems based on ITS amplification followed by sequencing for the identification of fungal pathogens in 26 culturally or histologically proven cases of IFD and one additional PCR positive case in FFPE tissue specimens. Molecular results from the FFPE samples were compared with the composite diagnosis according to the European Organization for Research and Treatment of Cancer (EORTC) criteria [13]. We especially aimed to analyze the quality of human and fungal DNA in FFPE samples and their level of age-dependent degradation, as well as the existence of PCR inhibition in these tissue samples.

## Methods

### Clinical specimens from patients with proven IFD

The University Hospital of Basel is a tertiary referral 950-bed institution serving north western Switzerland with a population of approximately half a million people. From 2003 to 2010 27 FFPE biopsy samples from sterile sites of 26 hospitalized patients with proven IFD were collected and retrospectively analyzed. The biopsies were all performed for diagnostic purpose without any link to our study. The study was approved by the local ethical committee (Ethische Komission beider Basel, EKBB). Proven IFD was defined by use of the EORTC criteria [13] on the basis of cultural or histopathological assessment of the tissue samples at the time the samples were collected. One sample which was PCR-positive from fresh tissue was added to the collection. The samples consisted of 21 lung, 2 skin, one cerebellum, one sinus sphenoidalis, one small intestine, and one soft tissue biopsy (Table 1).

**Table 1:** **Results of culture, histopathology, and PCR from fresh tissue compared to PCR from FFPE specimens (n = 27)**

Patient	Tissue site (storage in years)	Culture	Histopathology	ITS1-2PCR on fresh tissue	PCR on FFPE		Identification after sequencing (% identity)
					ITS1-2	ITS2	
*Culture positive samples*							
1	Lung (7)	*Aspergillus nidulans*	+	ND	+	+	*Hormographiella aspergillata* (100)
2	Lung (6)	*A. fumigatus/Fusarium sp.*	-	ND	-	+	*A. fumigatus* (99)
3	Lung (4)	*A. fumigatus*	+	ND	-	*+*	probably *A. lentulus* (100)
4	Lung (4)	*A. fumigatus*	+	ND	+	*+*	*A. fumigatus* (100)
5	Lung (4)	*A. fumigatus*	+	ND	-	+	*A. fumigatus* (99)
6	Lung (3)	*H. aspergillata* ^†^	+	ND	+	+	*H. aspergillata (100)*
7	Lung (5)	*A. fumigatus*	+	ND	-	+	*A. fumigatus* (99)
8	Lung (1)	*Rhizopus sp.*	+	ND	+	+	*Rhizopus microsporus/azygosporus (99)*
9	Skin (6)	*A. flavus*	ND	ND	+	+	*A. flavus* (100)
10	Skin (1)	*Alternaria* sp	ND	ND	+	+	*Alternaria sp.* (99)
11	Lung (4)	*A. nidulans*	+	ND	+	+	*A. nidulans* (100)
12	Lung (2)	*A. fumigatus*	-	ND	-	-	-
13	Lung (2)	*H. aspergillata* ^†^	+	ND	-	-	-
14	Lung (5)	*A. fumigatus*	+	ND	-	-	-
15	Soft tissue (7)	*Rhizopus sp.*	+	ND	-	-	-
16	Cerebellum (3)	H. aspergillata*^†^	+	ND	-	-	-
17	Lung (4)	*A. flavus*	+	ND	-	-	-
*Histopathology-positive, culture-negative samples*							
18	Small intestine (2)	-	+	ND	+	+	*A. fumigatus* (100)
19	Sinus sphenoidalis (5)	-	+	ND	-	-	-
20	Lung (4)	-	+	ND	-	-	-
21	Lung (3)	-	+	ND	-	-	-
22	Lung (2)	-	+	ND	-	-	-
*PCR positive, culture-negative samples*							
23	Lung (1)	-	+	*A. flavus*	+	+	*A. flavus* (100)
24	Lung (1)	-	+	*H. aspergillata*	+	+	*H. apergillata* (99)
25	Lung (1)	-	-	*R. pusillus/tauricus*	-	-	-
26	Lung (1)	-	+	*R. pusillus/tauricus*	-	-	-
27	Lung (1)	-	+	*Lichtheimia corymbifera*	-	-	-

*grown in blood culture, ^†^case previously described [27].

+,positive; −, negative; ND, not done.

### Fungal culture

Fungal culture was performed on all 27 biopsy specimens using standard procedures [14]. Isolated fungal organisms were identified by standard phenotypic procedures [15]. Confirmatory molecular identification was performed using the MicroSeq™ D2 rDNA Fungal Identification System (Life technologies™, Rotkreuz, Switzerland) [16]. In addition to the sequence data base of MicroSeq, we compared our sequences using the BLAST analysis search (National Center of Biotechnology Information, Washington DC, www.ncbi.nlm.nih.gov/BLAST).

### Histopathology

All samples were obtained during procedures performed in an operation theater supplied with laminar airflow. The samples were transported in sterile containers and fixed in 4% buffered formalin. After paraffin-embedding, biopsies were cut into 4 μm sections, routinely stained with haematoxylin and eosin, elastic-Van Gieson and alcian blue periodic acid-Shiff and reviewed by a pathologist. If there were histological findings suggestive of a fungal infection Grocott’s methenamine silver staining was performed additionally.

### Panfungal PCR assay from FFPE biopsies

#### Deparaffinization and DNA extraction

Three 20 μm tissue slices from each tissue block were transferred to a sterile 1.5 ml vial. 1 ml of xylol was added and incubated for 5 minutes. Xylol was discarded by pipetting after a centrifugation step of 14 000 rpm during 1 minute. Then 1 ml ethanol was added and incubated at room temperature for 1 minute. The ethanol was discarded by pipetting after a centrifugation step as mentioned above. Deparaffinized tissue specimens were dried in a SpeedVac (Savant AES1010) for 20 minutes. DNA was extracted and purified using QIAamp Mini Kit (Qiagen, Hombrechtikon, Switzerland) according to the manufacturer’s instructions with the exception of an additional heating step of 95°C for 12 minutes after pipetting 200 μl AL buffer. All FFPE tissue samples were incubated over night in proteinase K and ATL buffer at 56°C. The elution step was performed with 100 μl AE buffer. Extracted DNA was stored at −20°C.

#### PCR amplification of ITS1-2 and ITS2 followed by sequencing

Panfungal PCR was performed using primers ITS5 (forward) and ITS4 (reverse) for amplification of ITS region 1 and 2 and primers ITS3 (forward) and ITS4 (reverse) for detection of ITS region 2 [6]. PCR was performed in a total volume of 30 μl consisting of 0.2 μm of each primer and 15 μl of 2× HotStarTaq Master Mix (Qiagen). Four different volumes of DNA extract (0.5, 2, 5, and 10 μl) per sample were analyzed. AE Buffer was added accordingly to the PCR premix to reach the final volume of 30 μl. The PCR amplification program included an initial denaturation at 95°C for 15 minutes, followed by 45 cycles of denaturation at 94°C for 30 seconds, annealing at 58°C for 1 minute, and extension at 72°C for 1 minute. Final elongation was at 72°C for 7 minutes. The PCR reactions were run on a PE 2400 or on a Veriti™ using the 9600 emulation mode (Life technologies™). The PCR products were visualized on a UV transilluminator after electrophoresis on precast 3% ReadyAgarose™ gels (Bio-Rad, Reinach, Switzerland).

The PCR products were purified with Microcon PCR Filter Units (Millipore, Zug, Switzerland) and were sequenced in both directions using the PCR primers and the BigDye® Terminator version1.1 Cycle Sequencing Kit (Life technologies™). After a purification step with DyeEx 2.0 Spin Kit (Qiagen), the sequencing products were detected in an ABI PRISM 3100 genetic analyzer (Life technologies™). The sequences were edited and aligned using the SeqMan sequence analysis software (DNASTAR, Madison, USA) and compared with reference sequences using the BLAST analysis search (National Center of Biotechnology Information, Washington DC, www.ncbi.nlm.nih.gov/BLAST) as well as the search tool of the Centraalbureau voor Schimmelcultures, Utrecht, The Netherlands (CBS) as follows: www.cbs.knaw.nl/collections/BioloMICSSequences.aspx.

### Inhibition control using *C. krusei*-spiking

Inhibition of PCR amplification was evaluated by spiking of 2 μl *C. krusei* DNA to each PCR premix including 2 μl of sample extract to reach a good visible PCR product after PCR of ITS2 region followed by agarose gel electrophoresis.

### Identification of contaminating DNA

Despite strict measures to prevent DNA contamination, we detected erratically small amounts of contaminating DNA in negative extraction controls during the development of our panfungal PCR assay. These fragments could be identified after sequencing as *Malassezia restricta/sympodialis* DNA.

Our criteria for a positive panfungal PCR result were two or more positive PCR reactions per clinical sample or a single reproducible PCR signal.

### PCR amplification of fragments of the beta-globin gene

To evaluate the quality of the extracted DNA from the FFPE biopsies, 2 μl and 0.5 μl from each DNA extract was used for multiplex PCR amplification resulting in 262 bp (primer pair KM29/KM38), 408 bp (primer pair GH20/GH21), and 860 bp (primer pair TI860F/TI860R) fragments of the beta-globin gene according to [5]. The same method for PCR and agarose gel analysis was used as described above for ITS detection with exception of the annealing step which was at 55°C for 30 sec. This analysis was applied to the 26 clinical specimens representing IFD and the PCR positive sample, but also to 10 FFPE control samples with no suspicion of IFD, which were stored at different time periods from 1 month up to 10 years.

### Panfungal PCR assay from fresh biopsies

Fresh biopsies were analyzed identically according to the FFPE samples with 2 exceptions. (1), Tissue lysis from fresh specimens was done at 56°C for up to 3 hours instead of overnight incubation, and (2), PCR amplification and direct sequencing was performed only with the ITS1-2 PCR system using primers ITS5 and ITS4 as described previously [17].

### Evaluation of analytical sensitivity

The sensitivity of our two panfungal PCR tests ITS1-2 and ITS2 was compared with fungal culture using *Candida krusei* reference strain ATCC14243 as described previously [18]. Briefly, aliquots of 10-fold serial dilutions of *C. krusei* suspensions were extracted according to the protocol for fresh tissue samples. Corresponding PCR signals measured after agarose gel electrophoresis were then compared with the number of *C. krusei* cells by culture calculating the amount of organisms per PCR amplification and per CFU/ml.

We also assessed the sensitivity of our broad-range fungal PCR assays with *Aspergillus*-specific PCR systems which are expected to be more sensitive. Therefore, both panfungal PCR assays (ITS1-2 and ITS2) were tested in an external pilot quality control study (ASPDNA10) from the European Quality Control for Molecular Diagnostics (QCMD) programme for the detection of *Aspergillus* sp. from blood specimens. The panel included 12 samples containing different amounts of *Aspergillus* DNA or conidia as well as negative controls.

## Results

### Patients with proven IFD

#### Overview

We analyzed a total of 26 specimens corresponding to patients with proven IFD and one PCR-positive sample (Table 1). Of these 27 specimens 17 (63.0%) were culture-positive, 5 (18.5%) histopathology-positive and culture-negative, and 5 (18.5%) were PCR-positive analyzing fresh biopsies but culture-negative. In all 5 samples with cultural or molecular detection of *Hormographiella aspergillata* the histopathological report was Aspergillus like dichotomous branching. The cases from patient 6, 13, and 16 with cultural detection of *H. aspergillata* have been published previously by Conen et al. [19] (Table 1).

#### PCR analysis of FFPE specimens

PCR amplification and identification in 14 (51.9%) of the 27 FFPE specimens were positive: 11 (64.7%) among the 17 culture positive samples, 1 from the 5 histologically positive, culture negative- and 2 among the 5 PCR-positive, culture-negative FFPE tissue samples (Table 1).

Organisms identified by PCR corresponded to the results of culture and of PCR from fresh tissues. We noticed two differing results; detection of *H. aspergillata* in the FFPE sample from patient no. 1 and *A. lentulus* in the sample from patient no. 3 from which *A. nidulans* and *A. fumigatus* were grown on culture respectively. In the first case we repeated the whole analysis including DNA extraction using a second aliquot from formalin-fixed tissue and could confirm the PCR result with a 100% identity to *H. aspergillata* reference strains. In the sample from patient no. 3 ITS2 PCR and sequencing resulted in a 349-basepair-long sequence with a 100% match to two *A. lentulus* reference sequences (accession no. EF669968, EF 669970). Because the ITS region is not discriminative enough to identify this organism to the species level, we designated our molecular result as “probably” *A. lentulus* [20]. In patient 8, the cultural finding of *Rhizopus* sp. has been improved by PCR to *R. microsporus/azygosporus*. Specification to the species level of *Alternaria* in the specimen from patient 10 was not possible with conventional identification procedures or with ITS-based PCR.

In the total of 27 FFPE samples ITS2- and ITS1-2 panfungal PCR were positive in 51.8% and 37% respectively.

As can be seen in Table 2, the best performance to achieve a positive PCR signal was the use of 0.5 μl DNA extract for ITS-2 PCR. The sequence lengths analyzed from ITS1-2 and ITS2 system were from 408 to 641, and 342 to 384 nucleotides, respectively. The quality of the sequences was generally high with one exception of the ITS1-2 fragment using 5 μl extract from patient 3 resulting in 115 nucleotides, allowing only identification as *Aspergillus* sp (see also Table 3).

**Table 2:** **PCR amplification from the different volumes of DNA extract of the 14 PCR-positive FFPE specimens**

Sample No	ITS1-2				ITS2				PCR-based identification
	10 μl	5 μl	2 μl	0.5 μl	10 μl	5 μl	2 μl	0.5 μl	
1	-	-	+	+	-	+	+	+	*H. aspergillata*
2	-	-	-	-	-	-	+	+	*A. fumigatus*
3	-	+*	-	-	-	-	+	+	Probably *A. lentulus*
4	-	-	-	+	-	-	+	+	*A. fumigatus*
5	-	-	-		-	-	-	+	*A. fumigatus*
6	-	-	+	+	+	+	-	+	*H. aspergillata*
7	-	-	-	-	-	-	+	+	*A. fumigatus*
8	-	-	-	-	-	-	-	+	*Rhizopus microsporus/azygosporus*
9	+	+	+	+	-	+	+	+	*A. flavus*
10	+	+	+	+	-	+	+	+	*Alternaria* spp
11	-	-	+	+	-	-	+	+	*A. nidulans*
18	-	-	+	-	-	+	+	+	*A. fumigatus*
23	-	-	+	-	-	+	+	+	*A.flavus*
24	-	-	+	-	-	+	+	+	*H. aspergillata*
**Total positive PCR signals**	2	2	9	6	1	6	11	14	

^+^,positive; −, negative.

*partial sequence identified as *Aspergillus* sp.

**Table 3:** **Overview of storage period, ß-globin PCR of FFPE DNA, and ITS-PCR-based fungal result**

Sample no	Storage in years	Spiking	ß-globin PCR [amplicon length,bp]		ITS-PCR-based identification (sequence length of ITS2 / ITS1-2 in bp)
			2 μl	0.5 μl	
1	7	+	(+)	(+) [262]	*H. aspergillata* (376 / 541)
15	7	-	-	-	-
2	6	-	-	-	*A. fumigatus* (355 / negative)
9	6	+	(+)	(+) [262]	*A. flavus* (349 / 528)
7	5	-	-	-	*A. fumigatus* (354 / negative)
19	5	-	-	-	-
14	5	-	-	-	-
17	4	-	-	-	-
11	4	-	-	(+) [262]	*A. nidulans* (355 / 416)
3	4	+	-	-	probably *A. lentulus* (349 / 115 partial)
4	4	+	-	(+) [262]	*A. fumigatus* (354 / 567)
5	4	+	-	(+) [262]	*A. fumigatus* (353 / negative)
20	4	+	-	(+) [262]	-
21	3	+	-	(+) [262]	-
16	3	+	+	+ [408]	-
6	3	+	+	+ [408]	*H. aspergillata* (383 / 641)
12	2	+	(+)	(+) [262]	-
18	2	+	+	+ [408]	*A. fumigatus* (350 / 177 partial)
22	2	+	+	+ [408]	-
13	2	-	(+)	(+) [262]	-
10	1	+	+	+ [408]	*Alternaria* sp. (346 / 585)
26	1	+	+	+ [408]	-
8	1	+	+	+ [408]	*Rhizopus microsporus/azygosporus* (372 / 628)
23	1	+	+	+ [408]	*A. flavus* (342 / 408)
25	1	+	+	+ [408]	-
24	1	+	+	+ [408]	*H. aspergillata* (384 / 554)
27	1	-	-	-	-

Bands after agarose gel electrophoresis: +, strong; (+), faint; −, absent.

### Quality of the extracted DNA from FFPE biopsies

We detected inhibition of PCR amplification using spiking with *C. krusei* DNA in about the half of the samples, suggesting that inhibiting substances might be co-eluted during extraction and purification of the FFPE samples (data not shown). As shown in Table 3, 17 of the 27 samples exhibited DNA degradation based on partial beta-globin gene amplification. A relation between DNA degradation and time of storage of the FFPE tissue specimens was observed. All specimens stored longer than 3 years showed DNA degradation. This phenomenon was also observed in the 10 control samples with no suspicion of IFD (data not shown). In contrast, our results of ITS-based detection of fungal organisms were not related to the beta-globin findings and consequently not dependent on the duration of FFPE storage; in 8 of 13 samples which were stored longer than 3 years and showing DNA degradation, a fungal organism could be identified (Table 3).

### Contaminating DNA

Contaminating DNA identified as *Malassezia restricta/sympodialis* was found in 6 PCR reactions from 5 different specimens. Identical DNA sequences also were rarely identified in our negative extraction controls. The 5 organisms *Torrubiella* sp, *Liphiostoma* sp., *Panellus stipticus*, *Davidiella* sp., and *Coniosporium* sp. were detected only in single PCR reactions originating from 5 samples and identification of these organisms could not be confirmed in a repeated testing.

### Analytical sensitivity

The analytical sensitivity compared to culture was 0.3 *Candida krusei* organisms per amplification, corresponding to 60 CFU/ml for both ITS-based PCR assays.

The results of the quality control panel from QCMD, Glasgow, Scotland for the detection of *Aspergillus* DNA including our panfungal PCR results are shown in Table 4. In comparison to the results of the 27 participating laboratories that performed genus-specific PCR tests, our two panfungal PCR assays showed a considerable proficiency.

**Table 4:** **Results of external quality control panel for detection of** ***Aspergillus*** **DNA from QCMD 2010**

Sample participants* No	Sample content	Sample matrix	Sample concentration	Fungal PCR		% correct results
				ITS1-2	ITS2	
01	*A. fumigatus* DNA	Plasma	30,000 G.eq/ml	+	+	88.9
04	*A. fumigatus* DNA	Plasma	3000 G.eq/ml	+	+	70.4
02	*A. fumigatus* DNA	Plasma	300 G.eq/ml	-	-	25.9
03	Clinical negative	Plasma		-	-	81.5
05	*A. fumigatus* DNA	TE buffer	30,000 G.eq/ml	+	+	88.9
07	*A. fumigatus* DNA	TE buffer	3000 G.eq/ml	-	-	48.1
09	*A. fumigatus* DNA	TE buffer	300 G.eq/ml	-	-	29.6
06	Analytical negative	TE buffer		-	-	77.8
13	*A. fumigatus* conidia	Plasma	1000 Con/ml	+	+	77.8
10	*A. fumigatus* conidia	Plasma	100 Con/ml	-	-	59.3
11	*A. fumigatus* conidia	Plasma	10 Con/ml	-	-	40.7
12	Clinical negative	Plasma		-	-	85.2

*n = 27, with permission of QCMD, Glasgow, Scotland; G.eq, Genome equivalent; Con, Conidia.

+, positive*, A. fumigatus*; −, negative.

## Discussion

Applying the panfungal PCR to 26 FFPE samples from patients with proven IFD and to one PCR-positive sample, we found a sensitivity of 53.8% in total and 64.7% in culture-positive specimens according to the EORTC criteria. The ITS2 PCR targeting the shorter fragment showed a higher detection rate with a sensitivity of 53.8% compared to the ITS1-2 fragment (sensitivity 38%). Both assays proved to display a high analytical sensitivity detecting 60 CFU/ml *C. krusei.* Moreover, the 2 tests reached a considerable result in an *Aspergillus* proficiency panel from QCMD intended for use in *Aspergillus*-specific PCR assays.

An important factor which may have influenced the sensitivity might be the composition of fungal organisms detected. Two studies clearly showed that the sensitivity is significantly elevated when yeasts and not only filamentous fungi were identified, suggesting that yeasts can be detected more easily in FFPE samples [9],[11]. Accordingly in the study of Rickerts et al. [11], the sensitivity dropped from 70% to 62% when the cases with yeasts were omitted.

Inhibition of PCR amplification in about one third of the samples - suggesting that inhibiting substances might be co-eluted during extraction and purification of the FFPE samples - might be an additional factor which could be the reason of the only moderate sensitivity of the PCR assays. As can be seen in Table 3 the PCR inhibition was mainly in correlation to the amplification of the human beta-globin gene. In a study of Provan et al. [21] a PCR product <300 base pairs could only be amplified in 60% of the DNA samples extracted, which suggests that the DNA contained PCR inhibitor and/or was degraded. Bretagne et al. [22] excluded 3 of 55 bronchoalveolar lavage samples as they did not allow correct amplification of the internal competitive control presumably because of PCR inhibition. Therefore, internal amplification controls are crucial for PCR analysis to rule out false negative results.

In a previous study we have analyzed the diagnostic yield of panfungal PCR on fresh tissue biopsy specimens; in contrast to FFPE specimens, we could show a high sensitivity of the PCR approach on patient level and a higher positive rate compared to culture and/or histology [17]. These results additionally support the finding that formaldehyde inhibits fungal PCR.

In our study, we used different volumes of sample DNA to improve the efficiency of the two conventional PCR assays. We showed that this approach was an important factor for accurate detection of fungal pathogens in FFPE samples including the identification of contaminating DNA.

In concordance to the studies of Muñoz-Cadavid et al. and Cabaret et al. [7],[9] we found a higher detection rate in the PCR system amplifying the shorter fragment, demonstrating degradation of fungal DNA in FFPE tissue. Muñoz-Cadavid et al. used identical primer pairs and found 38 and 65 positive samples for ITS1-2 and ITS2 PCR, respectively. The difference in performance between our two PCR assays may be caused by the effect of degradation, having greater influence on larger PCR applicants. Cabaret et al. analyzed 16 FFPE sinus fungal balls. Ten were positive using ITS-based PCR detecting a PCR fragment >300 bp, whereas mitochondrial PCR amplifying <150 bp was positive in 15 from 16 specimens. The authors consequently concluded that ITS-based identification cannot be used as a single detection system in FFPE samples with suspicion of IFD.

We observed a high amount of FFPE samples with DNA degradation measured with beta-globin gene amplification. According to other studies [4],[5], the degradation of the human DNA was time dependent, with significant degradation in samples older than 3 years. Crosslinking between nucleic acid and proteins might be another reason, resulting in base pair lengths of approximately 200 bp or less, as this has been described in samples treated with formaldehyde [23]. On the contrary, PCR amplification including correct identification of fungal DNA was astonishingly not age-dependent and, to our knowledge, is reported for the first time in this study (Table 3). A possible explanation for this finding could be a protective effect of the tenacious fungal cell wall against formalin.

In our series a discrepant PCR result was detected compared to culture; *H. aspergillata* was detected on molecular level in the FFPE lung tissue from patient 1 which had been stored for 7 years and in which *A. nidulans* had been detected in culture. Infection with *H. aspergillata* has been reported from several critically ill patients [24]-[26], including from our institution describing the cases from patient 6, 13, and 16 in this study [27]. Antifungal therapy options are limited as there is limited activity of caspofungin and none of fluconazole [27]. Our patient was successfully treated with voriconazole. Retrospectively, mixed or concomitant infection in patient 1 could have been present which might not have been detected by the conventional diagnostic methods; pre-treatment with antifungals may lower the culture yield and fungal elements in tissue samples may often appear as pleomorphic, impeding diagnosis of mixed infections and representing diagnostic challenge.

In patient no. 2 the fungal cultures revealed *A. fumigatus* and *Fusarium sp*. Here, only *A. fumigatus* could be detected by PCR. This might be due to preferential amplification of individual amplicons by the ITS-assay, depending on PCR conditions and primer mismatch, in samples where different templates are present; a phenomenon which is described in 16S rRNA gene-targeting bacterial community analysis [28].

A further inconsistent finding was the molecular identification of probable *A. lentulus* in patient 3 with the cultural result of *A. fumigatus*.

*A. lentulus*, a new species of *Aspergillus,* that has morphological characteristics poorly distinguishable from *A. fumigatus* [29], was originally described by Balajee *et al*. in 2005 [20]. Correct species identification only can be accomplished by sequencing of the *beta-tubulin* and *rodlet A* gene. A characteristic of *A. lentulus* is its reduced susceptibility to multiple antifungal drugs [29]-[31]. Interestingly, in this patient’s lung lesions were progressive under therapy with voriconazole. We now hypothesize that in 2006 the patient was infected with *A. lentulus,* which could not correctly been identified morphologically, and that inefficient antifungal therapy for the causative *A. lentulus* may have allowed progression. As can be seen in a similar study based on ITS1 PCR from Lau et al. [8], this organism was detected in further 5 cases.

### Limitation and strengths

First, the added value of our results for the identification of fungi in culture negative samples is only limited and the sample size small. Second, the assays were only applied to tissue samples and not tested on less invasive sample types like bronchoalveolar lavage. However, the study was conducted to test the performance of the assays against proven cases of IFD according to EORCT criteria (with the exception of sample 25) and to test the accuracy of identification of fungal pathogens in FFPE samples.

Furthermore the overall methodology of the study might only be satisfactory because of a fairly low sensitivity of 53.8%. However we have studied limiting factors of performing panfungal PCR on FFPE tissues in a meticulous way and have reviewed patient’s clinical histories in detail, especially in cases where PCR results differed from cultural results.

## Conclusions

To conclude, panfungal diagnostic PCR assays applied to FFPE specimens provide accurate identification of molds in highly degraded tissue samples and correct identification in samples stored up to 7 years despite limitations in sensitivity mainly caused by PCR inhibition and DNA degradation of formalin. One of the main disadvantages of molecular approaches in FFPE specimens might be the contamination with ubiquitous environmental fungi. However, in combination with conventional laboratory test methods panfungal PCR may increase diagnostic yield, especially in culture-negative samples and might complement conventional diagnostic tests particularly in cases of mixed infections. The added value for the identification of culture-negative samples though is limited in this study and studies with higher sample sizes are necessary.
